# The independent predictive value of admission serum ferritin concentration for prognosis in elderly patients with community-acquired pneumonia in the emergency department

**DOI:** 10.3389/fcimb.2024.1505207

**Published:** 2025-01-10

**Authors:** Xiangqun Zhang, Na Shang, Da Zhang, Junyuan Wu, Shubin Guo

**Affiliations:** ^1^ Emergency Medicine Clinical Research Center, Beijing Chao-Yang Hospital, Capital Medical University, Beijing, China; ^2^ Beijing Key Laboratory of Cardiopulmonary Cerebral Resuscitation, Beijing Chaoyang Hospital, Capital Medical University, Beijing, China; ^3^ Clinical Center for Medicine in Acute Infection, Capital Medical University, Beijing, China

**Keywords:** ferritin, community-acquired pneumonia, elderly patients, prognostic prediction, emergency department (ED)

## Abstract

**Background:**

Community-acquired pneumonia (CAP) poses a significant health threat to the elderly population, leading to high morbidity and mortality rates. Serum ferritin, a critical indicator of iron metabolism, plays a pivotal role in inflammation and immune regulation. Nevertheless, its specific prognostic relevance in elderly patients with CAP remains unclear. This study aimed to evaluate the predictive capacity of serum ferritin in determining the prognosis of elderly patients with CAP and to investigate its effectiveness when combined with the sequential organ failure assessment (SOFA) or CURB-65 (confusion, uremia, respiratory rate, blood pressure, aged ≥65 years) scores.

**Methods:**

This retrospective cohort study included 451 elderly patients (aged ≥65 years) diagnosed with CAP according to established criteria. Serum ferritin concentrations were measured upon admission and various prognostic indicators such as 28-day mortality, mechanical ventilation requirement, and vasopressor administration were analyzed in conjunction with white blood count (WBC), C-reactive protein (CRP), procalcitonin (PCT), lactate (Lac), SOFA scores, and CURB-65 scores. The independent predictive value of ferritin was assessed through receiver operating characteristic (ROC) curve analysis and multivariate logistic regression.

**Results:**

Among the 451 patients, 99 (22%) died within 28 days. The area under the curve (AUC) of serum ferritin for predicting 28-day mortality was 0.75 (95%CI: 0.695-0.805). Ferritin outperformed WBC, CRP, and PCT in predictive performance, and its performance was comparable to Lac. When combined with SOFA or CURB-65 scores, the AUC of ferritin for predicting 28-day mortality increased to 0.84 and 0.847, respectively (*P*<0.001). Moreover, the AUC of ferritin for predicting vasopressor administration was 0.707, which increased to 0.864 and 0.822 when combined with SOFA or CURB-65 scores, respectively (*P*<0.001). Ferritin could predict mechanical ventilation requirement with an AUC of 0.618, but it was not an independent risk factor, and its predictive ability was not significantly different from other indicators.

**Conclusion:**

Admission serum ferritin is an independent predictor for the prognosis of elderly patients with CAP, and it exhibits a strong ability to predict the 28-day mortality and vasopressor administration. The combination of ferritin with SOFA and CURB-65 scores significantly improves the prognostic predictive potency.

## Introduction

Community-acquired pneumonia (CAP) is an acute disease characterized by high morbidity and mortality (Cillóniz et al., 2018). The elderly population, due to age-related physiological changes and a greater prevalence of comorbidities, faces an elevated risk of morbidity and mortality associated with CAP (Brown et al., 2018). Rapid progression of the disease in elderly patients often necessitates hospitalization and can lead to poor clinical outcomes in the absence of timely or appropriate treatment. Therefore, identifying reliable prognostic markers is of great significance for predicting disease severity and developing personalized treatment strategies, thus improving the clinical outcomes of patients with CAP (Metlay et al., 2019).

As a protein that can store iron in the cells, ferritin has been extensively explored in different clinical conditions, and it can fulfill various functions, not limited to iron metabolism. Considered an acute-phase reactive protein, it has shown a significant increase in concentration during systemic inflammatory reactions, serving as an indicator of immune system dysregulation severit (Kernan and Carcillo, 2017). The elevated serum ferritin concentration has been validated to be associated with a poor prognosis in inflammatory diseases (such as sepsis and other critical illnesses) (Fang et al., 2022), and this indicator also plays a predictive role in assessing the severity and mortality of diseases. For example, a higher ferritin concentration is associated with the increased mortality of patients with sepsis (Horvat et al., 2022), and it is also correlated with a poor prognosis of some tumors and autoimmune diseases (Wang et al., 2023; Giemza-Stokłosa et al., 2019). Despite the established role of ferritin in various diseases, its predictive value in elderly patients with CAP has not been fully clarified. Therefore, it is necessary to investigate the potential of ferritin as a prognostic tool in elderly patients with CAP, especially in predicting some key outcomes. Given these facts, this study was conducted to assess the predictive ability of ferritin concentrations (the 28-day mortality, mechanical ventilation requirement, and vasopressor administration) in elderly patients with CAP and identify whether ferritin can be used as a reliable biomarker in clinical practice. These efforts are expected to improve the prognostic prediction of CAP and guide clinical decisions for the treatment of this disease.

## Materials and methods

### Subjects

A retrospective study was conducted on a cohort of 451 elderly patients diagnosed with CAP who were admitted to the emergency department (including the emergency rescue room, emergency observation room, and emergency intensive care unit) of Beijing Chao-yang Hospital, Capital Medical University from 2021 to 2024. The study received approval from the Human Research Ethics Committee of Beijing Chao-yang Hospital, Capital Medical University.

### Diagnostic criteria

The diagnosis of CAP was based on the identification of new infiltrative shadows on chest radiographs in conjunction with at least one of the following symptoms: (1) cough; (2) expectoration; (3) dyspnea; (4) body temperature >38.0°C (5) abnormal breath sounds or rales upon auscultation (Niederman et al., 2001).

Inclusion and Exclusion Criteria:

In this study, the inclusion criteria included: (1) patients aged ≥65 years; (2) patients who met the diagnostic criteria of CAP. The exclusion criteria included: (1) patients with advanced disease, including malignant tumors (advanced or metastatic tumors) and end-stage liver disease or kidney disease; (2) patients who were hospitalized within 14 days before the occurrence of symptoms; (3) patients with cystic fibrosis, active pulmonary tuberculosis, and severe immunosuppression; (4) patients who required vasopressors and mechanical ventilation at the time of admission.

### Grouping

In this study, the results were evaluated via a 28-day follow-up study. Vasopressor administration, (non-invasive/invasive) mechanical ventilation requirement, and 28-day mortality were selected as endpoint events. A total of 451 elderly patients with CAP (including 268 males and 183 females) were included in this study. Based on the occurrence of endpoint events, 99 patients were classified into the death group, while 352 patients were allocated to the survival group. Furthermore, 110 patients were categorized into the mechanical ventilation group, with 341 patients belonging to the non-mechanical ventilation group. Additionally, 124 patients were segmented into the vasopressor administration group, and 327 patients were included in the non-vasopressor administration group.

### Data collection

The demographic characteristics, including age and gender, as well as vital signs and medical history, were systematically documented for all participants. Blood samples, totaling 5-10 mL, were obtained within 6 hours of patient admission and promptly placed in heparin-containing tubes. Subsequent analyses included white blood count (WBC), blood gas assessment, and blood biochemistry tests, all conducted within 24 hours of blood collection. The severity of illness was quantified using the SOFA and CURB-65 scores, derived from clinical indicators and laboratory parameters.

### Laboratory tests

The automated hematology detection analyzer (Sysmex XS-500i, Sysmex Corporation Kobe, Japan) was used to perform the WBC analysis. Plasma lactate (Lac) levels were assessed using a blood gas analyzer (GEM Premier 3000, Instrumentation Laboratory, Lexington, MA, USA), with the normal range established as 0.7-2.5 mmol/L. The serum procalcitonin (PCT) concentration was measured using the Mini-VIDAS immunoanalyzer of BioMerieux (Block Scientific, Bohemia), with the limit of detection (LOD) being 0.05ng/L. The serum C-reactive protein (CRP) concentration was determined by immunoturbidimetry (BNII, Siemens Healthcare Diagnostics Inc., Germany), with the normal range being 0-10ng/mL. The serum ferritin concentration was tested by chemiluminescence immunoassay (Beckman Coulter DXI800, USA), with the normal range defined as 11-306.8ng/mL.

### Statistical analysis

The statistical analysis was performed with the aid of SPSS 16.0 (SPSS Inc., Chicago, IL, USA). The normally distributed data were expressed as mean ± standard deviation (SD), and the non-normally distributed data were expressed as median (P25, P75). The Mann-Whitney U test was deployed for between-group comparisons, and the Kruskal-Wallis univariate analysis was employed for comparisons involving multiple groups. The WBC, Lac, PCT, CRP, ferritin, SOFA scores, and CURB-65 scores were analyzed in the analysis of vasopressor administration, mechanical ventilation requirement, and 28-day mortality. Additionally, the ROC curve was plotted, and the AUC, sensitivity, and specificity were calculated. Compared with AUC, the Z value was calculated as per Z=(A1-A2)/(SE12+SE22) 1/2 (Z0.05 = 1.96, Z0.01 = 2.58). The binary logistic regression analysis was conducted to identify the independent predictors of vasopressor administration, mechanical ventilation requirement, and 28-day mortality. All statistical analyses were performed based on two-tailed tests. P<0.05 was considered statistically significant.

## Results

### Baseline data of patients with CAP

A total of 547 elderly patients with CAP met the inclusion criteria and provided written informed consent upon admission to the emergency department. According to the medical records, 59 patients refused mechanical ventilation, 3 patients refused vasopressor administration (coinciding with mechanical ventilation), and 37 patients were lost to follow-up after being transferred to other hospitals. Eventually, 451 patients completed the 28-day follow-up study. The baseline data of these CAP patients are listed in **Table 1**.

**Table 1:** Baseline characteristics of elderly patients with community-acquired pneumonia (CAP).

Characteristics	Non-Vasopressor	Vasopressor	*P*	Non-Mechanical Ventilation	Mechanical Ventilation	*P*	Survivor	Non-Survivors	*P*
	(n=327)	(n=124)		(n=110)	(n=341)		(n=352)	(n=99)	
Age, years	72(66-83)	81 (68-85)	<0.001	72 (66-83)	79 (68-84)	0.044	72 (66-82)	82(70-86)	<0.001
Male, %	59.6	59.5	0,883	59.4	59.1	0.953	60.5	55.6	0.376
COPD, %	11	40	0.313	7.4	23.9	<0.001	10.2	11.7	0.671
CVD, %	41.5	45	0.441	39.4	43.6	0.445	41.7	45.9	0.453
Hypertension, %	51.3	44.8	0.798	25.5	34.6	0.075	29.3	33.2	0.46
DM, %	40.6	47.9	0.179	42.1	43.6	0.783	41.1	47.5	0.258
CHF, %	4.3	6.7	0.341	3.8	8.3	0.064	4	8.2	0.094
Tumor, %	18.2	22.3	0.392	18.9	20	0.806	17.8	24.2	0.149
MBP, mmHg	98 (87-113)	96 (73-112)	<0.001	99 (84-111)	93 (79-117)	0.544	98 (85-112)	98 (76-115)	0.014
Respiratory rate, beats/min	20 (19-24)	20 (19-27)	0.408	20 (18-23)	22 (20-28)	0.175	20 (19-23)	21 (19-28)	0.917
Temperature, °C	37 (36-37)	36 (36-37)	0.303	37 (36-37)	37 (36-37)	0.649	37 (36-37)	37 (36-37)	0.233
Heart rate, beats/min	90 (78-102)	97 (85-113)	0.147	92 (81-105)	96(82-113)	0.049	91 (81-105)	96 (83-113)	0.089
WBC, Í10^9^/L	8 (6.9-11.1)	10.7(4.6-15.4)	<0.001	12.7 (7.7-18.4)	15.5 (7.8-15.7)	0.003	7.9 (5.7-11.3)	11.9 (6.4-19.6)	0.005
CRP	15.7(1.4-30.6)	29.9 (6-121.7)	<0.001	13.5 (1.5-35.2	34.9(6.9-126)	0.002	13.3 (1.4-30.6)	46.3(11-168.4)	<0.001
PCT, ng/mL	0.07(0.05-2.02)	0.81 (0.05-6.19)	<0.001	0.07(0.05-2.19)	0.66 (0.05-6.07)	0.009	0.07(0.05-1.8)	1.57 (0.05-6.19)	<0.001
Lac, mmol/L	1.1(0.7-1.5)	3.9 (1.5-8.5)	<0.001	1 (0.7-1.7)	2.8 (1.8-5.3)	<0.001	1.1(0.7-1.5)	5.8 (2.1-10.2)	<0.001
Ferritin	188.3(100-425.6)	442.7(182.8-1269.1)	<0.001	202.8(111.7-452.7)	383.7(127.1-943.7)	<0.001	190.2(98.35-437.2)	464(247.7-1324.5)	<0.001
CURB-65	2 (1-2)	3(2-4)	<0.001	2 (1-2)	3 (2-4)	<0.001	2 (1-2)	3 (2-4)	<0.001
SOFA score	4(1-7)	10 (8-13)	<0.001	5 (2-8)	9 (5-11)	<0.001	5 (2-8)	10 (8-13)	<0.001

COPD, chronic obstructive pulmonary disease; CVD, Cerebrovascular disease; DM, Diabetes mellitus; CHF, chronic heart failure; MBP, myelin basic protein; Lac, lactate; PCT, procalcitonin; WBC, white blood cell; CRP, C-reactive protein; qSOFA, quick sequential organ failure assessment; SOFA, sequential organ failure assessment; CURB-65, confusion, urea, respiratory rate, blood pressure and age.

The 451 elderly patients with CAP were divided into the vasopressor administration group (N=124) and the non-vasopressor administration group (N=327) according to the based on their vasopressor administration in the 28-day follow-up study. The statistical analysis results indicated that there were significant differences in the age (*P*<0.001), mean arterial pressure (MBP) (*P*=<0.001), WBC, CRP, PCT, Lac, ferritin (*P*<0.001), SOFA scores, and CURB65 scores (*P*<0.001) of patients between the two groups (**Table 1**). These findings indicate that older age, elevated biomarkers (e.g., ferritin, CRP, and PCT), and higher SOFA and CURB-65 scores are strongly associated with vasopressor requirements, reflecting greater disease severity.

Besides, these patients were divided into the mechanical ventilation group (N=110) and the non-mechanical ventilation group (N=341) according to the application of ventilators in the 28-day follow-up study. The statistical analysis results suggested that there were significant differences in the age (*P*=0.044), chronic obstructive pulmonary disease (COPD) (*P*<0.001), heart rate (HR) (*P*=0.049), WBC (*P*=0.003), CRP (*P*=0.002), PCT (*P*=0.009), Lac, ferritin (*P*<0.001), SOFA scores, and CURB65 scores (*P*<0.001) of patients between the two groups (**Table 1**). This indicates that factors such as pre-existing COPD, systemic inflammation (elevated ferritin, CRP, and PCT levels), and higher SOFA/CURB-65 scores are predictive of mechanical ventilation requirements, emphasizing the importance of these indicators in clinical decision-making.

Moreover, these patients were also divided into the death group (N=352) and the survival group (N=99) according to their survival in the 28-day follow-up study. The statistical analysis results indicated that there were significant differences in the age (*P*<0.001), WBC (*P*=0.005), MBP (*P*=0.014), CRP, PCT, Lac, ferritin (*P*<0.001), SOFA scores, and CURB65 scores (*P*<0.001) of patients between the two groups (**Table 1**). These findings highlight that advanced age, elevated inflammatory markers (e.g., ferritin, CRP, PCT), and higher severity scores (SOFA and CURB-65) are closely linked to poor prognosis, including higher 28-day mortality rates.

### Correlation of serum ferritin and other indicators

To investigate the relationship between serum ferritin levels and various clinical markers including WBC, CRP, PCT, Lac, SOFA scores, and CURB65 scores, the Spearman correlation analysis was performed (**Table 2**). The analysis revealed significant correlations between serum ferritin and WBC (r=0.311, *P*<0.001), CRP (r=0.339, *P*<0.001), PCT (r=0.176, *P*<0.001), Lac (r=0.177, *P*<0.001), SOFA scores (r=0.286, *P*<0.001), and CURB65 scores (r=0.312, *P*<0.001 (**Table 2**). These results indicate that ferritin levels are significantly associated with markers of inflammation and disease severity, reinforcing its potential as an integrative marker for assessing the overall health status of elderly CAP patients.

**Table 2:** The correlation between serum ferritin and other indicator.

Variables	WBC	Lac	PCT	CRP	SOFA	CURB65
Spearman correlation	0.311	0.177	0.176	0.339	0.286	0.312
*P*	<.001	<.001	<.001	<.001	<.001	<.001

### Logistic regression analysis of each variable for the prognosis of patients with CAP

In this study, the independent predictors were analyzed using multivariate logistic regression, as shown in **Table 3**. The results indicated that serum ferritin (*P*=0.003, OR=1.001, 95%CI: 1.001-1.002), WBC (*P*=0.035, OR=1.049, 95%CI: 1.003-1.079), Lac (*P*<0.019, OR=1.027, 95%CI: 1.004-1.049), SOFA scores (*P*<0.001, OR=1.23, 95%CI: 1.145-1.321), and CURB65 scores (*P*<0.001, OR=2.686, 95%CI: 1.905-3.788) were independent risk factors for the 28-day mortality in elderly patients with CAP (**Table 3A**). Besides, serum ferritin (*P*=0.034, OR=1.001, 95%CI: 1-1.001), WBC (*P*=0.043, OR=1.048, 95%CI: 1.001-1.096), PCT (*P*=0.032, OR=1.062, 95%CI: 1.005-1.122), Lac (*P*=0.026, OR=1.026, 95%CI: 1.003-1.049), SOFA scores (*P*<0.001, OR=1.35, 95%CI: 1.25-1.458), and CURB65 scores (*P*<0.001, OR=2.682, 95%CI: 1.912-3.762) were independent risk factors for the vasopressor administration in elderly patients with CAP (**Table 3B**). Additionally, SOFA scores (*P*=0.006, OR=1.081, 95%CI: 1.023-1.143) and CURB65 scores (*P*<0.001, OR=1.626, 95%CI: 1.241-2.131) were independent risk factors for the mechanical ventilation requirement in elderly patients with CAP (**Table 3C**).

**Table 3A:** Binary logistic regression analysis of 28-day mortality in elderly patients with community-acquired pneumonia (CAP).

Variables	*β*	*S.E*	Wald	*P*	OR (95% CI)
Ferritin	0.001	0	8.773	0.003	1.001 (1-1.002)
WBC	0.048	0.023	4.423	0.035	1.049 (1.003-1.097)
CRP	0.001	0.002	0.456	0.499	1.001 (0.997-1.006)
PCT	0.024	0.029	0.695	0.404	1.025 (0.967-1.085)
Lac	0.026	0.011	5.508	0.019	1.027 (1.004-1.049)
SOFA	0.207	0.036	32.217	<.001	1.23 (1.145-1.321)
CURB65	0.988	0.175	31.784	<.001	2.686 (1.905-3.788)
Constant	-6.221	0.576	116.67	<.001	

**Table 3B:** Binary logistic regression analysis of vasopressor use in elderly patients with community-acquired pneumonia (CAP).

Variables	*β*	*S.E*	Wald	*P*	OR (95% CI)
Ferritin	0.001	0	4.478	0.034	1.001 (1-1.001)
WBC	0.046	0.023	4.083	0.043	1.048 (1.001-1.096)
CRP	-0.001	0.002	0.16	0.69	0.999 (0.995-1.003)
PCT	0.06	0.028	4.582	0.032	1.062 (1.005-1.122)
Lac	0.026	0.011	4.961	0.026	1.026 (1.003-1.049)
SOFA	0.3	0.039	58.463	<.001	1.350 (1.250-1.458)
CURB65	0.987	0.173	32.634	<.001	2.682 (1.912-3.762)
Constant	-6.184	0.572	116.956	<.001	

**Table 3C:** Binary logistic regression analysis of mechanical ventilation use in elderly patients with community-acquired pneumonia (CAP).

Variables	*β*	*S.E*	Wald	*P*	OR (95% CI)
Ferritin	0	0	2.845	0.092	1 (1-1.001)
WBC	0.013	0.02	0.404	0.525	1.013 (0.974-1.053)
CRP	0.001	0.002	0.258	0.611	1.001 (0.997-1.004)
PCT	-0.01	0.023	0.191	0.662	0.99 (0.946-1.036)
Lac	0.005	0.009	0.376	0.54	1.005 (0.988-1.023)
SOFA	0.078	0.028	7.685	0.006	1.081 (1.023-1.143)
CURB65	0.486	0.138	12.452	<.001	1.626 (1.241-2.131)
Constant	-3.028	0.343	77.793	<.001	

### Prediction of the prognosis of elderly patients with CAP

**Figure 1:**
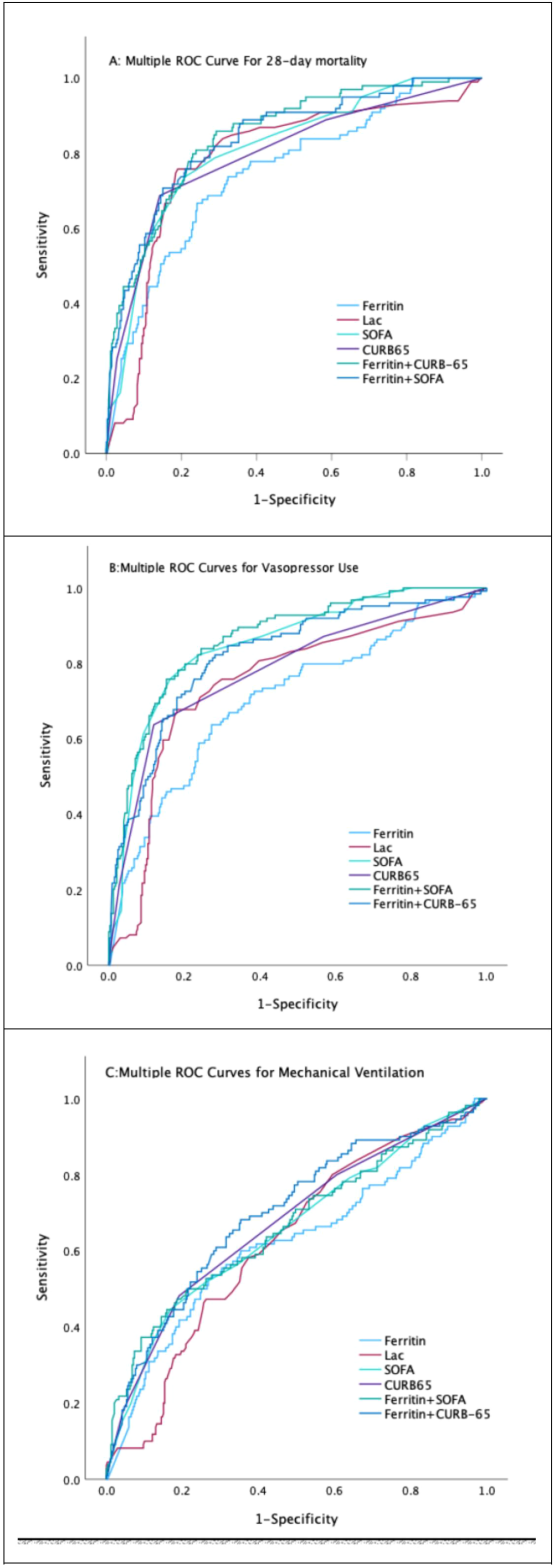
ROC Curves of Various Predictors for Prognostic Outcomes in Elderly Patients with CAP.

The findings pertaining to the serum ferritin levels and other associated variables, as well as their amalgamations, in the context of forecasting the 28-day mortality, administration of vasopressors, and mechanical ventilation requirement among elderly patients with CAP are presented in **Table 4** and illustrated in **Figure 1**.

**Table 4A:** Characteristics of predictors for 28-day mortality in elderly patients with CAP.

Variables	AUC (95% CI)	*S.E*	*P*	Cut off	Sensitivity	Specificity
Ferritin	0.75 (0.695-0.805)	0.028	<0.001	380.4	66.7	75.9
WBC	0.666 (0.599-0.733)	0.034	<0.001	11.25	45.5	79.3
CRP	0.673 (0.610-0.737)	0.032	<0.001	26.55	61.6	69.6
PCT	0.658 (0.597-0.719)	0.031	<0.001	0.305	66.7	64.8
Lac	0.785 (0.731-0.839)	0.028	<0.001	2.55	75.8	81
SOFA	0.812 (0.764-0.86)	0.024	<0.001	7.5	77.7	79
CURB-65	0.798 (0.744-0.852)	0.028	<0.001	2.5	68.7	85.8
Ferritin+SOFA	0.84 (0.795-0.885)	0.023	<0.001	0.255	70.7	84.9
Ferritin+CURB-65	0.847 (0.804-0.89)	0.022	<0.001	0.178	80.8	76.1

**Table 4B:** Characteristics of predictors for vasopressor use in elderly patients with CAP.

Variables	AUC (95% CI)	*S.E*	*P*	Cut off	Sensitivity	Specificity
Ferritin	0.707 (0.651-0.763)	0.028	<0.001	327.9	63.7	72.8
WBC	0.636 (0.573-0.699)	0.032	<0.001	11.05	50	79.2
CRP	0.634 (0.573-0.694)	0.031	<0.001	37.25	46.8	78.6
PCT	0.655 (0.598-0.712)	0.029	<0.001	0.305	64.5	66.4
Lac	0.748 (0.694-0.802)	0.027	<0.001	2.45	67.7	82
SOFA	0.851 (0.811-0.89)	0.02	<0.001	7.5	75.8	83.8
CURB-65	0.784 (0.733-0.835)	0.026	<0.001	2.5	63.7	88.1
Ferritin+SOFA	0.864 (0.827-0.902)	0.019	<0.001	0.3	75.4	84.7
Ferritin+CURB-65	0.822 (0.777-0.867)	0.023	<0.001	0.207	82.3	71.9

**Table 4C:** Characteristics of predictors for mechanical ventilation in elderly patients with CAP.

Variables	AUC (95% CI)	*S.E*	*P*	Cut off	Sensitivity	Specificity
Ferritin	0.618 (0.553-0.683)	0.033	<0.001	381.8	52.7	73.2
WBC	0.594 (0.531-0.657)	0.032	0.003	11.65	40	78.5
CRP	0.596 (0.532-0.660)	0.033	0.004	39.65	44.5	77.6
PCT	0.578 (0.517-.639)	0.031	0.012	0.14	62.7	55
Lac	0.623 (0.565-0.682)	0.03	<0.001	2.55	52.7	73.8
SOFA	0.657 (0.596-0.719)	0.031	<0.001	8.5	55.5	83.2
CURB-65	0.674 (0.613-0.734)	0.031	<0.001	2.5	48.2	80.9
Ferritin+SOFA	0.665 (0.602-0.728)	0.032	<0.001	0.278	50	78.8
Ferritin+CURB-65	0.697 (0.638-0.755)	0.03	<0.001	0.214	68.2	64.7

WBC, white blood cell; CRP, C-reactive protein; PCT, procalcitonin; Lac, lactate; SOFA, sequential organ failure assessment; CURB-65, confusion, urea, respiratory rate, blood pressure and age.

The AUC of serum ferritin, WBC, CRP, PCT, Lac, SOFA scores, and CURB-65 scores for predicting the 28-day mortality in elderly patients with CAP was 0.75 (95%CI: 0.695-0.805, *P*<0.001), 0.666 (95%CI: 0.599-0.733, *P*<0.001), 0.673 (95%CI: 0.610-0.737, *P*<0.001), 0.658 (95%CI: 0.597-0.719, *P*<0.001), 0.785 (95%CI: 0.731-0.839, *P*<0.001), 0.812 (95%CI: 0.764-0.86, *P*<0.001), and 0.798 (95%CI: 0.744-0.852, *P*<0.001),respectively. The AUC of ferritin+SOFA and ferritin+CURB65 for predicting the 28-day mortality in elderly patients with CAP was 0.84 (95%CI: 0.795-0.855, *P*<0.001) and 0.847 (95%CI: 0.804-0.89, *P*<0.001), respectively. These results indicate that serum ferritin has predictive power for 28-day mortality, combining ferritin with SOFA and CURB-65 scores significantly enhanced the AUC to 0.84 (95%CI: 0.795-0.855, P<0.001) and 0.847 (95%CI: 0.804-0.89, P<0.001), respectively, suggesting that ferritin can improve the prognostic accuracy of these established scoring systems (**Table 4A**).

The AUC of serum ferritin, WBC, CRP, PCT, Lac, SOFA scores, and CURB-65 scores for predicting vasopressor administration in elderly patients with CAP was 0.707 (95%CI: 0.651-0.763, *P*<0.001), 0.636 (95%CI: 0.573-0.669, *P*<0.001), 0.634 (95%CI: 0.573-0.694, *P*<0.001), 0.655 (95%CI: 0.598-0.712, *P*<0.001), 0.748 (95%CI: 0.694-0.802, *P*<0.001), 0.851 (95%CI: 0.811-0.89, *P*<0.001), and 0.784 (95%CI: 0.733-0.845, *P*<0.001), respectively. The AUC of ferritin+SOFA and ferritin+CURB65 for predicting the vasopressor administration in elderly patients with CAP was 0.864 (95%CI: 0.827-0.902, *P*<0.001) and 0.822 (95%CI: 0.777-0.867, *P*<0.001), respectively. These findings highlight that serum ferritin provides good predictive accuracy for vasopressor use, comparable to Lac and superior to traditional markers like WBC and CRP. Combining ferritin with SOFA (AUC=0.864, 95%CI: 0.827-0.902, P<0.001) or CURB-65 (AUC=0.822, 95%CI: 0.777-0.867, P<0.001) further improves its predictive performance, underscoring the utility of ferritin in guiding critical care interventions (**Table 4B**).

The AUC of serum ferritin, WBC, CRP, PCT, Lac, SOFA scores, and CURB-65 scores for predicting the mechanical ventilation requirement in elderly patients with CAP was 0.618 (95%CI: 0.532-0.66, *P*<0.001), 0.594 (95%CI: 0.531-0.657, *P*=0.003), 0.596 (95%CI: 0.573-0.694, *P*=0.004), 0.578 (95%CI: 0.517-0.639, *P*=0.012), 0.623 (95%CI: 0.565-0.682, *P*<0.001), 0.657 (95%CI: 0.596-0.719, *P*<0.001), and 0.674 (95%CI: 0.617-0.734, *P*<0.001), respectively. The AUC of ferritin+SOFA and ferritin+CURB65 for predicting the mechanical ventilation requirement in elderly patients with CAP was 0.665 (95%CI: 0.602-0.728, *P*<0.001) and 0.697 (95%CI: 0.638-0.755, *P*<0.001), respectively. While ferritin exhibited limited predictive performance for mechanical ventilation (AUC=0.618), Combining ferritin with SOFA (AUC=0.665, 95%CI: 0.602-0.728, P<0.001) or CURB-65 (AUC=0.697, 95%CI: 0.638-0.755, P<0.001) provided modest improvements (**Table 4C**).

**Figure 2:**
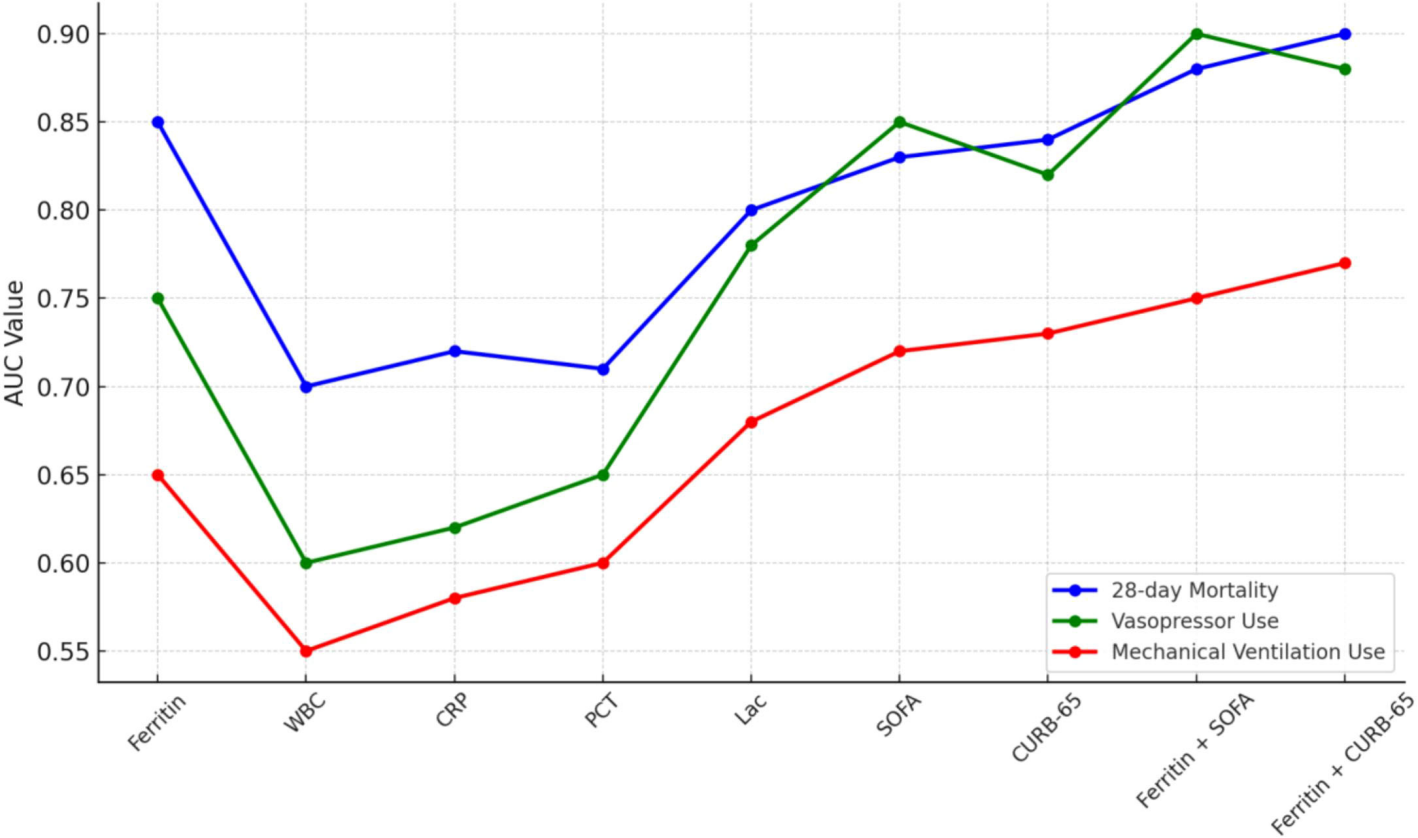
AUC comparison for ferritin and other indicators.

### Comparison of serum ferritin and other indicators in predicting the prognosis of elderly patients with CAP

The ability of serum ferritin and other indicators to predict the prognosis of elderly patients with CAP was compared based on their AUC values (**Table 5**; **Figure 2**). The results indicated that serum ferritin was superior to WBC (Z=2.278, *P*=0.022), CRP (Z=1.925, *P*=0.045), and PCT (Z=2.347, *P*=0.019) in predicting the 28-day mortality of these elderly patients with CAP. This finding highlights ferritin’s stronger prognostic value compared to traditional inflammatory markers, emphasizing its potential as a primary biomarker for mortality prediction. However, no statistically significant differences were found between serum ferritin and Lactate (Z=0.884, P=0.377), SOFA (Z=1.681, P=0.093), and CURB-65 (Z=1.212, P=0.225), indicating that ferritin performs comparably to these well-established severity scores in predicting 28-day mortality. Furthermore, both ferritin+SOFA and ferritin+CURB-65 exhibited superior predictive power for 28-day mortality compared to serum ferritin alone (Z=2.484, P=0.013; Z=2.724, P=0.006), suggesting that combining ferritin with severity scores significantly enhances prognostic accuracy (**Table 5A**).

**Table 5A:** For 28 day mortality (FerritinAUC 0.75).

Indicator	AUC	Z-value	P-value
WBC	0.666	2.278	0.022
CRP	0.673	1.925	0.045
PCT	0.658	2.347	0.019
Lac	0.785	0.884	0.377
SOFA	0.812	1.681	0.093
CURB-65	0.798	1.212	0.225
Ferritin + SOFA	0.84	2.484	0.013
Ferritin + CURB-65	0.847	2.724	0.006

**Table 5B:** For vasopressor use (FerritinAUC 0.707).

Indicator	AUC	Z-value	P-value
WBC	0.636	1.67	0.095
CRP	0.634	1.747	0.081
PCT	0.655	1.29	0.197
Lac	0.748	1.054	0.292
SOFA	0.851	4.185	< 0.001
CURB-65	0.784	2.015	0.044
Ferritin + SOFA	0.864	4.644	< 0.001
Ferritin + CURB-65	0.822	3.174	0.002

**Table 5C:** For mechanical ventilation use (FerritinAUC 0.618).

Indicator	AUC	Z-value	P-value
WBC	0.594	0.522	0.602
CRP	0.657	0.471	0.637
PCT	0.578	0.833	0.377
Lac	0.623	0.112	0.911
SOFA	0.657	1.131	0.258
CURB-65	0.674	1.24	0.216
Ferritin + SOFA	0.665	1.022	0.307
Ferritin + CURB-65	0.697	1.771	0.076

For vasopressor administration, serum ferritin demonstrated comparable predictive performance to WBC (Z=1.67, P=0.095), CRP (Z=1.747, P=0.081), and PCT (Z=1.29, P=0.197), indicating that ferritin performs similarly to traditional markers for this outcome. However, Lac (Z=1.054, P=0.292), SOFA (Z=4.185, P<0.001), CURB-65 (Z=2.015, P=0.044), ferritin+SOFA (Z=4.644, P<0.001), and ferritin+CURB-65 (Z=3.174, P=0.002) exhibited superior predictive accuracy compared to serum ferritin alone. These results suggest that combining ferritin with SOFA or CURB-65 scores enhances its ability to guide clinical decisions regarding vasopressor use (**Table 5B**).

For predicting mechanical ventilation requirements, serum ferritin did not significantly differ from other biomarkers, including WBC (Z=0.522, P=0.602), CRP (Z=0.471, P=0.637), PCT (Z=0.833, P=0.377), Lac (Z=0.112, P=0.911), SOFA (Z=1.131, P=0.258), CURB-65 (Z=1.24, P=0.216), ferritin+SOFA (Z=1.022, P=0.307), and ferritin+CURB-65 (Z=1.771, P=0.076). This indicates that ferritin has limited predictive value for mechanical ventilation, and its performance is comparable to traditional inflammatory markers (**Table 5C**).

## Discussion

In this study, the prognostic value of serum ferritin in elderly patients with community-acquired pneumonia (CAP) was comprehensively analyzed. The findings demonstrated that serum ferritin is an independent predictor for 28-day mortality, the need for vasopressor administration, and mechanical ventilation in this patient population. Furthermore, combining serum ferritin with established clinical scoring systems, such as CURB-65 and SOFA, significantly enhanced the ability to predict these outcomes, underscoring its utility in guiding risk stratification and optimizing clinical management for elderly CAP patients.

Ferritin has demonstrated superior predictive performance for 28-day mortality in elderly CAP patients compared to other inflammatory markers such as CRP and PCT. Specifically, ferritin exhibited statistically significant differences in AUC values for predicting 28-day mortality compared to CRP (Z=1.925, P=0.045) and PCT (Z=2.347, P=0.019). CRP and PCT are among the most commonly used biomarkers in clinical practice for CAP and are well-recognized prognostic indicators for the disease (Self et al., 2017; Liu et al., 2023). Both markers are independent predictors of CAP outcomes (Khan et al., 2017; Self et al., 2016; Hussain et al., 2022), with elevated levels closely associated with the host’s inflammatory response to infection mm. In bacterial pneumonia, daily monitoring of PCT levels is an effective and safe tool for guiding antibiotic duration and cessation (Branche et al., 2019) (Schuetz et al., 2017). However, as nonspecific acute-phase reactants, CRP and PCT levels may also rise in sterile inflammatory conditions such as pancreatitis, major surgery, or cardiopulmonary resuscitation, limiting their specificity (Carbonell et al., 2023). The superior prognostic capability of ferritin compared to CRP and PCT underscores its value as a more reliable biomarker in predicting CAP outcomes. Notably, ferritin’s predictive performance was comparable to established scoring systems, such as SOFA and CURB-65 scores, in predicting 28-day mortality (P>0.05). This suggests that ferritin, as an independent prognostic marker, has predictive potency comparable to established scoring systems.

The combination of ferritin with CURB-65 or SOFA scores also showed superior performance in predicting the vasopressor administration in these patients. The AUC of ferritin for predicting the vasopressor administration in these patients was 0.707, but in conjunction with CURB-65 or SOFA scores, the AUC rose significantly to 0.822 (*P*<0.001) and 0.864 (*P*<0.001), respectively. These results further supported that ferritin was an effective prognostic biomarker. In particular, the application of ferritin in combination with clinical scoring systems can significantly improve the prediction accuracy.

The predictive performance of ferritin for mechanical ventilation was less pronounced, with an AUC of 0.618. However, when combined with CURB-65 scores, the AUC improved to 0.697. This lower predictive efficacy for mechanical ventilation may reflect the complexity of patient decision-making in elderly CAP cases, where personal preferences or family choices often play a role in accepting or declining invasive treatments. Such factors may limit the clinical utility of ferritin in forecasting the need for mechanical ventilation.

Ferritin, as the main iron storage protein in the human body, plays a fundamental role in regulating iron metabolism and immune responses. It consists of 24 subunits and can store up to 4,500 iron ions, ensuring iron homeostasis through storage and release mechanisms (Yanatori et al., 2023). In normal physiological conditions, ferritin levels accurately reflect the body’s iron reserves. However, during pathological states such as infection, ferritin functions as an acute-phase reactant protein, limiting pathogen access to iron—an essential nutrient for microbial growth—through a process termed “nutritional immunity” (Marchetti et al., 2020; Gerner et al., 2020). This iron regulation is complemented by transferrin, which transports free iron in the bloodstream, maintaining systemic iron balance and supporting immune defense

In addition, ferritin helps mitigate oxidative stress by regulating free iron availability. Free ferrous iron (Fe²^+^) can generate reactive oxygen species (ROS) through the Fenton reaction, leading to cellular damage (Nakamura et al., 2019; Gensluckner et al., 2024). The H subunit of ferritin exhibits ferric oxidase activity, converting Fe²^+^ to ferric iron (Fe³^+^), which is safely stored within the protein structure. This mechanism protects host cells from oxidative damage while maintaining antibacterial defenses (Pham et al., 2004). Escalated ferritin levels during infection are indicative of perturbed iron metabolism and an activated inflammatory response, both of which are hallmarks of severe CAP (Fonseca et al., 2023; Moreira et al., 2020).

Research has consistently supported the predictive value of serum ferritin in predicting all-cause mortality, particularly in critically ill patients and those with sepsis (Fang et al., 2022; Nandy et al., 2021; Lungu et al., 2024). Moreover, serum ferritin levels have been found to be closely linked to adverse outcomes in various conditions, including tumors, connective tissue diseases, and COVID-19 (Kirino et al., 2018; Plano et al., 2023; Wang et al., 2023; Perottet et al., 2022; Giemza-Stokłosa et al., 2019). Despite this, there is a scarcity of clinical studies on the predictive capacity of ferritin for CAP in elderly individuals. Mehta P reported that a ferritin concentration exceeding 1380 ng/mL indicated a poor prognosis in adults with acute respiratory distress syndrome (ARDS). Among 847 ARDS patients who received ventilator-assisted ventilation, 204 (24.1%) patients had a ferritin concentration of >1380 ng/mL and a mortality rate of 60%; the remaining patients had a ferritin concentration of <1380 ng/mL and a mortality rate of 35%. In addition, patients with a high ferritin concentration were found to have a significantly lower probability of weaning from mechanical ventilation than those with a low ferritin concentration (Mehta et al., 2024). In a study conducted by Goldhaber G, the association between ferritinemia (the serum ferritin concentration >1000 ug/L) and mortality among elderly inpatients was examined. The findings revealed a median survival time of merely 4.7 months for the 242 patients under scrutiny, with nearly 70% succumbing within a two-year period. Notably, the risk of mortality rose by 1.5% for each 100-unit elevation of serum ferritin concentration (Goldhaber et al., 2020). As reported in some studies, the serum ferritin concentration is significantly related to the survival rate of patients with acute exacerbation of idiopathic pulmonary fibrosis. Patients with a higher ferritin concentration usually have a lower survival rate. In particular, when the ferritin concentration is higher than 500 ng/mL, the risk of death increases significantly (Enomoto et al., 2018). The current study complements these findings by establishing practical thresholds specific to elderly CAP patients: ferritin levels exceeding 380.35 ng/mL for 28-day mortality, 327.85 ng/mL for vasopressor administration, and 381.75 ng/mL for mechanical ventilation. These thresholds provide clinicians with actionable benchmarks for early intervention. Furthermore, ferritin was an independent risk factor for the 28-day mortality and vasopressor administration, and there was no significant difference in the predictive ability for the 28-day mortality between ferritin and Lac in these elderly patients with CAP. Lac is a well-established predictor of both sepsis and CAP (Gattinoni et al., 2019; Huang et al., 2023; Frenzen et al., 2018), serving as an independent prognostic factor. Similarly, both ferritin and lactate have been identified as independent predictors, demonstrating comparable predictive performance for 28-day mortality and vasopressor use. The similar predictive ability of ferritin highlights its potential as a useful adjunct or alternative biomarker, particularly in clinical settings where lactate measurement may be less feasible.

In a neonatal study, it was observed that an increased ferritin concentration exceeding 248.5 ng/mL correlated significantly with a heightened risk of sepsis in newborns (Lungu et al., 2024). Furthermore, multiple studies have demonstrated a noteworthy rise in mortality rates for patients with sepsis and COVID-19 when their ferritin concentrations ranged from 135 ng/mL to 821.5 ng/mL (Fang et al., 2022; Tonial et al., 2021; Cihakova et al., 2021; Liu and Liu, 2023). The variation in the specified cutoff value could likely stem from differences in subjects’ ages and experimental methodologies. This investigation’s findings emphasize the clinical importance of considering ferritin thresholds alongside patient-specific factors to achieve more precise prognostication and targeted care.

The present study highlights the importance of incorporating ferritin into established scoring systems, such as SOFA and CURB-65, to enhance predictive accuracy for CAP outcomes. The integration of ferritin-based thresholds enables clinicians to refine risk stratification models, allowing for more precise prognostic assessments and tailored treatment strategies. In resource-limited settings, ferritin’s affordability and accessibility make it a particularly valuable tool for guiding decisions in emergency and critical care scenarios.

Nevertheless, there are still some limitations in this study. Firstly, the cut-off values proposed in the continuous retrospective single-center study need validation in an independent and multicenter study cohort. Secondly, dynamic monitoring of ferritin was not conducted, and such data could have informed treatment decisions. Thirdly, the retrospective clinical study was unable to sufficiently identify cases where mechanical ventilation was indicated but refused by patients or their families, potentially affecting the accuracy of predicting the need for mechanical ventilation in elderly patients with CAP. Finally, the 2007 IDSA/ATS CAP guidelines recommended by the American Thoracic Society and Infectious Diseases Society of America[3] were not included in the analysis due to missing data. This scoring system has been shown to provide a more accurate assessment of high-risk status in CAP. Future research should address these gaps by employing prospective, multicenter designs and incorporating real-time monitoring of ferritin levels to validate its dynamic role in predicting CAP outcomes

## Conclusion

Admission serum ferritin is an independent predictor for the prognosis of elderly patients with CAP, and it exhibits a strong ability to predict the 28-day mortality and vasopressor administration in these patients. The combination of serum ferritin and SOFA or CURB-65 scores can significantly improve the predictive potency. Based on the results of this study, it is recommended that serum ferritin should be used as an important biomarker for risk assessment in elderly patients with CAP, thus better identifying high-risk patients and formulating personalized treatment protocols.

## Data Availability

The raw data supporting the conclusions of this article will be made available by the authors, without undue reservation.
